# Light-Emitting Textiles: Device Architectures, Working Principles, and Applications

**DOI:** 10.3390/mi12060652

**Published:** 2021-06-02

**Authors:** Marco Cinquino, Carmela Tania Prontera, Marco Pugliese, Roberto Giannuzzi, Daniela Taurino, Giuseppe Gigli, Vincenzo Maiorano

**Affiliations:** 1 Department of Mathematics and Physics “Ennio De Giorgi”, University of Salento, via Arnesano, 73100 Lecce, Italy; marco.cinquino@unisalento.it (M.C.); marco.pugliese@unisalento.it (M.P.); roberto.giannuzzi@unisalento.it (R.G.); daniela.taurino@unisalento.it (D.T.); giuseppe.gigli@unisalento.it (G.G.); 2 Institute of Nanotechnology (CNR-NANOTEC), National Research Council, via Monteroni, 73100 Lecce, Italy; vincenzo.maiorano@nanotec.cnr.it

**Keywords:** light-emitting e-textiles, alternating current electroluminescent devices, light emitting diodes, light electrochemical cells, polymeric optical fibers

## Abstract

E-textiles represent an emerging technology aiming toward the development of fabric with augmented functionalities, enabling the integration of displays, sensors, and other electronic components into textiles. Healthcare, protective clothing, fashion, and sports are a few examples application areas of e-textiles. Light-emitting textiles can have different applications: sensing, fashion, visual communication, light therapy, etc. Light emission can be integrated with textiles in different ways: fabricating light-emitting fibers and planar light-emitting textiles or employing side-emitting polymer optical fibers (POFs) coupled with light-emitting diodes (LEDs). Different kinds of technology have been investigated: alternating current electroluminescent devices (ACELs), inorganic and organic LEDs, and light-emitting electrochemical cells (LECs). The different device working principles and architectures are discussed in this review, highlighting the most relevant aspects and the possible approaches for their integration with textiles. Regarding POFs, the methodology to obtain side emissions and the critical aspects for their integration into textiles are discussed in this review. The main applications of light-emitting fabrics are illustrated, demonstrating that LEDs, alone or coupled with POFs, represent the most robust technology. On the other hand, OLEDs (Organic LEDs) are very promising for the future of light-emitting fabrics, but some issues still need to be addressed.

## 1. Introduction

In recent years, the growing innovations in the field of wearable technologies have been changing and improving our everyday lives. Typical examples of wearable and portable devices are in the form of smart watches, glasses, wristbands, and belts. Unfortunately, these kinds of devices are composed of rigid and planar materials, so they may be uncomfortable if worn. In this context, the possibility to integrate microelectronic devices with fabrics represents an important innovation toward the development of a more comfortable and versatile technology [1]. In particular, fabric represents a ubiquitous element of our life and therefore it is the ideal platform for portable and wearable device integration. The idea is to combine the typical textile characteristics such as flexibility, lighter weight, wearability, breathability, and low cost with electronic functionalities. Different sensors have been already integrated into textiles such as pressure sensors [2], strain sensors [3], human stress sensors based on electrochemical transistors [4], glucose electrochemical sensors [5], etc. Power generators and energy storage devices are also fundamental for the advancement of wearable technology. Triboelectric nanogenerators [6], piezoelectric nanogenerators [7], and solar cells based on dyes [8], organic materials [9], and perovskites [10] have been successfully developed. Energy storage devices such as batteries [11] and supercapacitors [12,13,14,15] have also been fabricated on textiles.

Light emission is another functionality that can be integrated with textiles with great potential as a visual communication element useful in sensing applications, fashion design, and light therapy. Flexibility, reasonable lifetimes, washability, large display areas, and low production costs are certainly the fundamental requirements for obtaining light-emitting fabrics that are competitive on the market. All these aspects are discussed in this review, and the issues to solve for the future of light-emitting textiles are highlighted. In particular, light emission can be integrated with textiles in different ways: employing self-emitting devices or using optical fibers coupled with external light-emitting devices, such as LEDs. Self-emitting devices can be integrated directly onto the fiber or can be fabricated and/or attached on top of the fabric surface. Different device architectures have been employed, whose working principles, advantages, disadvantages, challenges related to textile integration, and literature results are summarized in Section 2. In Section 2, the optical fibers approach is also described. In Section 3 and Section 4, the possible health and environmental concerns related with such technologies and the main application areas are discussed.

## 2. Light Emitting Technologies: Working Principles and Textile Integration

### 2.1. ACEL Devices

**Figure 1:**
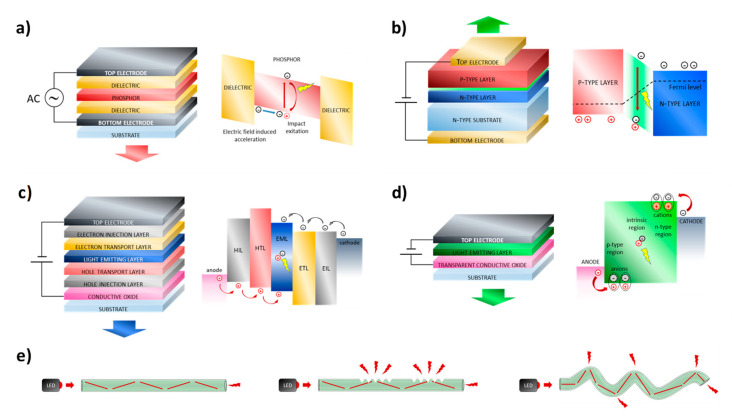
Device architecture and working principle of (**a**) ACEL device; (**b**) LED device; (**c**) OLED device; (**d**) LEC device; (**e**) Schematic representation of distal-end-emitting optical fibers and side-emitting optical fibers by using the microperforation and the macrobending approaches.

The phenomenon of alternating current electroluminescence (ACEL) was reported for the first time by Destriau in 1935 [16]. He observed that copper-doped zinc sulfide crystals, suspended in castor oil and sandwiched between two mica sheets, emitted light with the application of a strong alternating electric field. Only in 1952, after the development of transparent conducting electrodes based on tin oxide (SnO_2_), the first practical device was prepared [17]. It consisted of a doped ZnS powder embedded in a dielectric matrix and sandwiched between two electrodes. Today, ACEL devices are fabricated in two different ways: in the simplest approach, the dielectric and the phosphor are mixed together and then deposited as a single layer; alternatively, the phosphor can be deposited as thin film and sandwiched between two dielectrics, as reported in Figure 1a [18]. In both cases, from the electrical point of view, the structure of the device is that of a capacitor. The working principle of this kind of device involves different processes: electron injection, electron transport, electron impact and excitation, and finally radiative recombination (Figure 1a) [19]. Specifically, when a high AC voltage is applied, a high electric field is generated between the electrodes. If the electric field is sufficiently high, the electrons at the dielectric-phosphor interface can be injected into the emitting layer (hot injection). The electrons are then accelerated by the electric field toward the opposite electrode, hitting and exciting the phosphor particles. The following radiative relaxation process gives rise to visible light emission. Different phosphor materials can be used and different colors can be obtained by changing the doping impurities. Such devices are usually fabricated by using solution-based deposition techniques such as screen printing in ambient conditions, showing great potential for large-area displays. Considering the working principle, different typologies of electrodes can be used with at least one of the two being transparent without stringent requisite in terms of work function. The total thickness of the device is in the order of tens of microns and such a characteristic confers sufficient flexibility of the device but also good mechanical stability. Regarding the optoelectronic properties, ACEL devices show a wide viewing angle, fast response time, low power consumption, and high contrast in addition to simple manufacturing and cost-effectiveness [18]. On the other hand, such devices show limited luminance values and the high AC voltage values usually employed (50–200 V) can be too hazardous for wearable applications. Furthermore, the ACEL device can be damaged by humidity and for such reason an encapsulation process is necessary [20].

**Figure 2:**
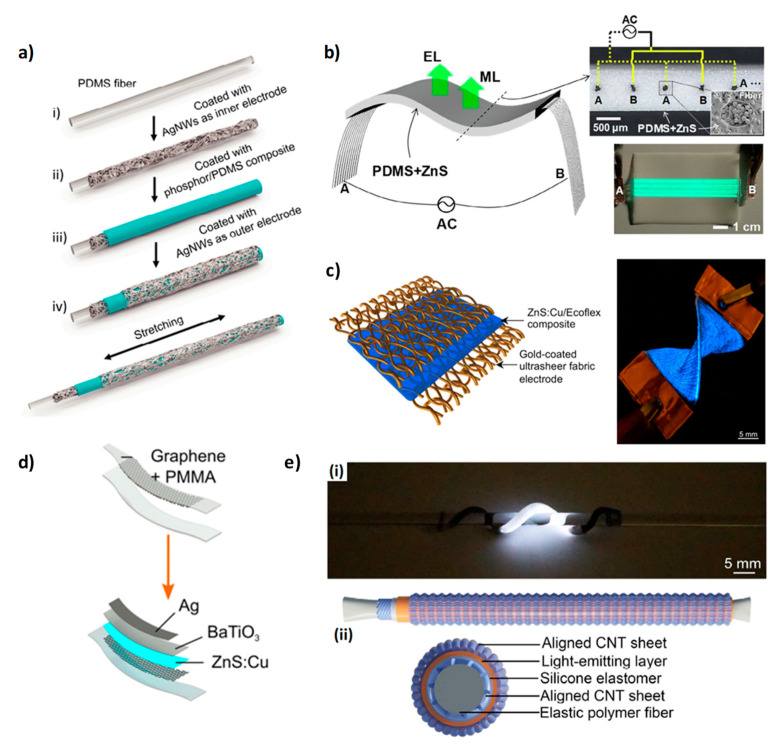
(**a**) Scheme of the fabrication process of a stretchable ACEL fiber. (**b**) Picture of a textile-fiber-embedded ACEL device, optical and scanning electron microscope cross-sectional images, and picture of the ACEL device under 500 V at 1 KHz. (**c**) Device structure based on ZnS:Cu/Ecoflex composite sandwiched between two gold-coated ultrasheer fabric electrodes and photograph of the light-emitting textile. (**d**) Graphene-based ACEL device. (**e**) Picture of a fiber-based ACEL device wrapped around a glass bar and scheme of the device structure. (**a**) Reproduced under the terms of the CC BY 4.0 license [27]. (**b**) Reproduced with permission [31]. Copyright 2020, Elsevier. (**c**) Reproduced with permission [32]. Copyright 2020, Elsevier. (**d**) Reproduced under the terms of the CC BY 4.0 license [30]. (**e**) Reproduced with permission [28]. Copyright 2018, Royal Society of Chemistry.

Examples of ACEL devices integrated on both single fibers and fabrics are discussed below and they are summarized in Table 1 and Table 2. One of the first examples of ACEL devices on single fibers that can be integrated with woven and knitted fabrics was demonstrated in 2012 [21]. The device consisted of a conductive yarn as the electrode, an insulating layer, an EL phosphor layer, and a second electrode based on a conductive yarn helically wound. An automatic dispensing and curing process was employed for the deposition of the insulating and emitting layer. The electro-optical characteristics of the light-emitting fiber are poor and need to be optimized, but the authors reported a high throughput fabrication process of ACEL devices on fiber. Another example of a fiber-based ACEL device created by using a scalable deposition technique was reported by researchers of Drexel University [22,23,24]. It consists of a slot-die coating system customized for a single fiber. The resulting devices were tested under different mechanical conditions and the authors demonstrated an improvement in strength and robustness when the devices were integrated into a knitted structure [24]. ACEL devices on flexible polyethylene terephthalate (PET) single fibers were also fabricated by dip-coating techniques and an AgNWs solution was employed for the transparent electrode deposition [25]. The device shows a luminance of about 200 cd/m^2^ at 195 V and 2 kHz with excellent flexibility, good mechanical characteristics, and wearability. The substitution of the material employed for the fiber fabrication can give an added value to the device. Flexible and water dissolvable PVA (poly(vinyl alcohol)) fibers were used for ACEL devices that can completely disintegrate after 180 min of water immersion with great potential in the reduction of e-waste [26]. A stretchable ACEL device (Figure 2a) was demonstrated by using a PDMS polydimethylsiloxane (PDMS) fiber with luminance values that do not change with stretching strain [27]. Stretchable single-fiber ACEL devices were also fabricated by the group of Peng by using two different methods [28,29]. The first one consists of 3D printing an elastomeric light-emitting tube composed of ZnS phosphor powders and silicon elastomer sandwiched between two aligned carbon nanotube sheets (Figure 2e) [28]. Such a device can be stretched up to 200% and shows a luminance of about 15 cd/m^2^ at 6.4 V/μm and 1500 Hz. The second method is based on the fabrication of a stretchable electroluminescent fiber via a continuous one-step extruding process. The fiber is composed of two inner hydrogel electrodes and a ZnS/silicon elastomer blend; it shows a luminance of about 250 cd/m^2^ at 8 V/μm and 1500 Hz when the 40% of ZnS is employed and it could be stretched up to 800% [29]. Graphene is another possible transparent electrode that can be interesting for light-emitting devices and the group of Craciun developed an ACEL device on top of tape-shaped graphene-covered fiber, the architecture of which is shown in Figure 2d [30].

As already mentioned in the introduction, the light-emitting device can also be fabricated directly on top of the fabric. ACEL devices were fabricated on fabric by using different deposition techniques and different transparent electrodes. Screen printing is the most used deposition technique for ACEL fabrication and it consists of transferring an ink onto a substrate through a mesh, changing the mesh to deposit different patterns. It was demonstrated that dispensing printing can be employed as an alternative deposition technique with comparable performance and with a versatility improvement since the deposition pattern can be changed digitally with a potential reduction of time and costs [33]. ACEL devices were also fabricated on semitransparent textiles (38% light transmission) by using a bottom emission configuration [34]. Inkjet printing and dispensing printing were employed to deposit poly(3,4-ethylenedioxythiophene (PEDOT:PSS) as a bottom transparent electrode and all the other layers, respectively. The device shows a maximum luminance of 44 cd/m^2^ at 400 V and 400 Hz. A bilayer of carbon nanotubes (CNTs) and PEDOT:PSS was also used as a bottom electrode for the fabrication of an ACEL device on top of a transparent PET mesh fabric [35]. CNTs were deposited via dip coating, while PEDOT:PSS was inkjet-printed and finally a nozzle extrusion system was employed for the deposition of dielectric and phosphor layers. The electroluminescent device reached a luminance of 75 cd/m^2^ at 200 V and 3.5 KHz. Other carbon nanomaterials were also employed as transparent electrodes [36,37]. For the deposition of the carbon nanomaterials (carbon nanotubes and graphene platelets), poly(methyl methacrylate) (PMMA) was first employed as polymeric carrier with poor results on the mechanical stability of the device. [36] More recently, a best result was reached by using a thermoplastic polyurethane polymer as an alternative carrier and the corresponding device had a luminance value of about 200 cd/m^2^ at 160 V and 1000 Hz and it did not exhibit visible degradation after 20 washing cycles [37]. An ACEL device was also successfully realized on top of a stretchable textile by using polypyrrole as a transparent conductive layer and a silicone elastomer as a matrix for the phosphor powder [38]. The resulting device had a luminance value of 350 cd/m^2^ that was preserved after hundreds of stretching cycles. An innovative approach for the fabrication of fabric-based ACEL devices was reported by the group of Carmichael [32,39]. They obtained a conductive stretchable textile by using a solution-based metallization process of knitted textile, demonstrating that the coating was conformal and the textile voids remained intact, thus preserving the stretchability [39]. The conductive knitted fabric showed a resistance reduction of 80% when the fabric was stretched to 15% and it maintained such resistance at 160% of strain; furthermore, its resistance was preserved after 10 washing cycles. The conductive substrate was tested as an electrode for ACEL devices by depositing on top of it BaTiO_3_+PDMS, ZnS:Cu+PDMS and PEDOT:PSS; a uniform blue emission was visible at 165 V and 37 kHz that was stable below 40% of strain [39]. An upgrade of this device architecture was recently reported and it consisted of a ZnS:Cu/Ecoflex composite sandwiched between two gold-coated fabric electrodes, as shown in Figure 2c. The device showed a uniform blue emission even after the application of a strain of 200% [32]. Another methodology for ACEL integration on textile was developed in 2019 and it can be considered a hybrid fiber/textile approach with improved mechanical stability [31]. The device consisted of Ag-coated nylon fibers embedded into a composite based on PDMS and ZnS phosphors (Figure 2b); when an AC voltage is applied the light is emitted from the ZnS particles that surround the fibers. The resulting coplanar structure showed high durability and stable electrical conductivity. 

From the results reported in this paragraph, it is evident that ACEL technology is robust enough for textile integration and high scalable deposition techniques can be employed for fabrication with the possibility of low-cost production. Although researchers are currently working to improve the potentiality of ACEL technology in the wearable electronic field [40], at the moment low luminance and high operating voltage represent the main factors limiting the development of this technology.

### 2.2. LED Devices

**Figure 3:**
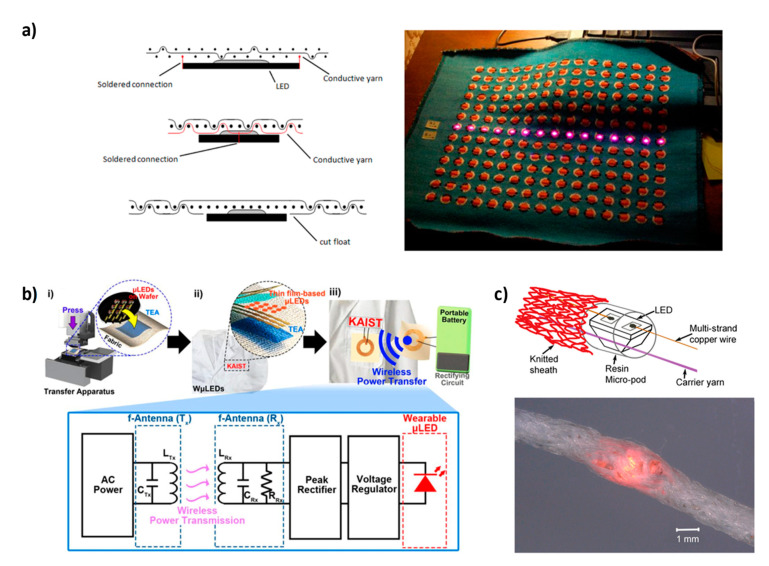
(**a**) Schematic illustration of LED integration with textile and picture of a textile LED display. (**b**) Representation of wireless-powered wearable μLEDs with a scheme of the circuit. (**c**) Scheme of the E-yarns structure with an LED and related picture. (**a**) Reproduced with permission [56]. Copyright 2013, NC State University. (**b**) Reproduced with permission [57]. Copyright 2018, Elsevier. (**c**) Reproduced under the terms of the CC BY 4.0 license [58].

Unlike ACEL devices, LEDs (light-emitting diodes) need a DC voltage of only a few volts to emit light. The first practical inorganic LED in the visible region was reported in early 1962 and it was based on Ga(As_1-x_P_x_) p-n junction [41]. A diode is a two-terminal electronic component that can conduct current only in one direction and, if specific light-emitting materials are employed, the current flux induces the formation of excitons that radiatively decay. Specifically, an LED is a p-n junction: when a voltage is applied, electrons and holes are pushed through the n and p regions, respectively, and they reach the “active region” that is close to the junction [42]. Electrons move into the conduction band, while holes move into the valence band; when electrons and holes recombine in the active region a radiative decay can occur, whose wavelength emission is related to the semiconductor band gap. A scheme of the working principle is shown in Figure 1b. Compounds based on Ga, Al, In, N, P, and As (III-V materials) are the core of inorganic LEDs [43]; such materials are crystalline in form and are usually deposited by using high temperature and high vacuum on sapphire substrates, which induce epitaxial growth. Since small defects can strongly affect the device characteristics and due to the high costs of the fabrication processes, the production of small-sized devices is usually more convenient. Nowadays, LEDs represent commercially available and high-performance light-emitting devices with dimensions of a few millimeters. However, LEDs do not exhibit the flexibility and stretchability properties desirable for textile integration. Nonetheless, this technology is reliable and numerous research efforts were made to integrate such devices with textiles. In particular, two different approaches are reported in the literature; the first approach involves the fabrication of a textile electronic circuit and the subsequent attachment of different electronic devices onto the circuit, including LEDs. Alternatively, the device can be directly embedded into the fiber; by using this approach, the device results are less visible to the viewer, the fabric is more comfortable, and the electrical interconnections are more mechanically robust. Regarding the first approach, two aspects need to be considered: the fabrication of the textile electrical circuits and their interconnection with external devices that can also be rigid. Different methods for the fabrication of electrical circuit have been reported in the literature: embroidering of conductive yarns [44,45], weaving [46,47] and knitting by using conductive and non-conductive yarns [48], laser cutting of conductive fabrics [49], screen printing [50], and use of plastic-based e-strips that can be woven like traditional yarns [51,52]. About the interconnection between the circuits and the external devices: soldering [49,51,52], gluing with conductive [46,52] and non-conductive adhesives [47], embroidering and stitching with conductive yarns [45,49], and crimping [53] are the most used techniques. In 2006, Buechley created an “e-textile construction kit” containing a microcontroller, sensors, and actuators (including LEDs), an infrared transceiver, an on/off switch, and a battery pack [44]. All the components were made of fabric or were designed to be easily stitched onto fabric; furthermore, the different elements can be connected through sewn conductive traces. The fabric circuit was obtained by using a methodology that combines the “iron-on” technique and laser cutting; firstly, a heat-activated paper adhesive is attached to a conductive fabric, afterward, a laser cutter is used to etch the circuit pattern into the fabric and the paper is removed from underneath the circuit, finally, the circuit is ironed onto the second piece of fabric [44,49]. The highly scalable and low-cost screen-printing technique was exploited by Kim et al. as a valid alternative to laser cutting, and the flip-chip method was employed to interconnect external electrical elements to the circuit [50]. The connection to the textile circuit can be also carried out by using a thermoplastic non-conductive adhesive [47,54]; by heat and pressure application the adhesive melts and electrical contact is formed. Stable mechanical and electrical contact is obtained after cooling. This method can be used with various types of textiles, and it represents a very easy and reliable approach. Package dies and more specifically LEDs can be also integrated via weaving. In particular, Parvoka et al. reported a specific weaving method able to integrate and hide the LED into the textile and this aspect is interesting to improve the aesthetics of the product (Figure 3a) [55,56]. Plastic-based e-fibers that can be woven into textiles by using a commercial manufacturing process were also reported [51]. The e-fibers were fabricated on top of a flexible plastic substrate by using standard microfabrication techniques and, afterward, the substrate was cut into 5-cm-long and <2-mm-wide strips. The obtained e-fibers were exposed to bending radii of 160 micron and tensile strain of about 20% without failure. In order to demonstrate the robustness of this approach, temperature and humidity sensors as well as LED devices were integrated on the e-fibers by soldering. A similar approach was recently used for the fabrication of e-strips on top of flexible plastic substrates, but in this case the weaving process was designed to conceal its presence from the wearer [52]. In particular, during the weaving process, bespoke pockets were obtained for the placement of the circuit; this methodology reduces the visibility of the circuit and makes it more comfortable when in contact with the skin. Also, in this case, external devices such as LEDs and microcontrollers were attached on top of the circuit by using a solder paste or anisotropic conductive paste. Moreover, the filament circuits embedded within the textile show better mechanical and durability performances compared to the corresponding filament that is not integrated into the fabric. 

MicroLEDs are an alternative to traditional LEDs for textile integration. They show high power efficiency, excellent stability, and their small dimensions do not compromise the flexibility and conformability of a typical textile. Lee et al. demonstrated a wireless-powered wearable microLED transferred on 100% cotton textile by using a transparent elastomeric adhesive, as shown in Figure 3b [57]. The device was resistant to stretching and bending, and it was also stable under high temperature and high humidity conditions (85 °C/85% RH).

As already mentioned, a different approach can be pursued in which the device is directly incorporated into the fiber. A thermal drawing process was reported in 2018 by Rein and coworkers [59]. This method is highly scalable and it allows for the creation of electrically connected diode fibers that maintain excellent performance after ten washing cycles. Another highly scalable method was reported by Hardy and coworkers, and it consists of a multistep process: soldering of dies onto a copper wire, encapsulation of the soldered die in a polymer micropod and finally twisting of textile yarns around the copper wire and the encapsulated dies (Figure 3c) [58,60,61]. Such a method was used for the embedding of both LEDs and photodiodes and it allows for a high-speed fabrication of e-yarns, which can be easily integrated into textiles by using the traditional equipment of the textile industry. 

Considering all the reported literature results, it is possible to conclude that LEDs are one of the best performing light-emitting technologies that can be integrated with textiles as a highly luminous, rigid, and point-like light source. Unfortunately, LEDs are not suitable for the fabrication of large-area and flexible light-emitting devices on textiles, and other technologies should be evaluated.

### 2.3. OLED Devices

OLEDs (Organic Light Emitting Diodes) are an evolution of LED devices in which the semiconductors are organic materials. The first OLED was fabricated by Tang and Van Slyke in 1987 and it was composed of two organic layers, an aromatic diamine as the hole transport layer and 8-hydroxyquinoline aluminum as an emissive layer, confined between indium tin oxide (ITO) and a Mg:Ag electrode [62]. In 1990, Burroughes et al. reported a high-efficiency green light-emitting polymer-based OLED by using poly(p-phenylene vinylene) as an emissive material [63]. To improve light emission efficiency, phosphorescent and thermally activated delayed fluorescent (TADF) materials have been developed and studied and nowadays they are largely applied as active materials in OLEDs [64,65,66]. The most employed deposition technique for OLED manufacturing is vacuum thermal evaporation, but organic materials can be easily processed by solution-based depositions, and for such reasons they can in principle be fabricated on a large area by using low-cost techniques, differently from inorganic LEDs. The deposition processes and the post-treatments of organic materials do not require high temperatures and it demonstrates the compatibility of such fabrication processes with flexible plastic substrates. Moreover, organic materials are intrinsically flexible, and this represents an advantage for flexible and textile-based device manufacturing in terms of mechanical and electrical stability. On the other hand, electrodes typically used in OLEDs, such as metals and ITO, can be damaged by mechanical deformations. Indeed, more flexible electrode materials are being studied [67]. Another important aspect of the fabrication of flexible and textile-based OLEDs is their thickness. OLEDs show a total thickness in the order of 100–200 nm, much thinner compared to ACEL devices and so each step of the fabrication process should be opportunely evaluated to avoid short circuits. In particular, the flatness of the substrate on which the device is deposited is a fundamental requirement. OLEDs for textile integration can be fabricated on single fibers or on top of textiles as planar devices. In the case of the integration of the light-emitting diode on a single fiber, it is sufficient to select a flexible and robust fiber with a smooth surface. On the other hand, planarization is mandatory to correctly fabricate a planar OLED on fabric because textiles show a waviness in the order of microns. Another important aspect is the high sensitivity of OLEDs to oxygen and water vapors that strongly reduce their lifetime. For such reasons, effective encapsulation is a crucial element.

**Figure 4:**
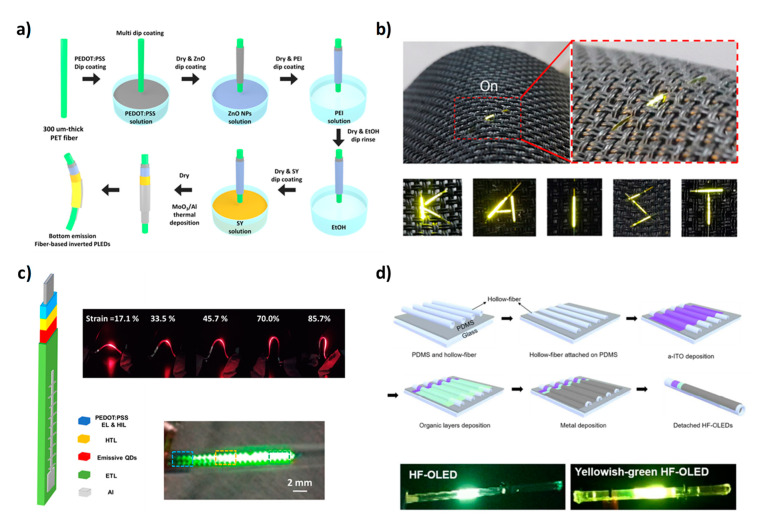
(**a**) Scheme of OLED fabrication on fibers. (**b**) Integration of a fiber-based OLED with a textile. (**c**) Device architecture of the quantum-dot light-emitting diode and pictures of the device in working conditions. (**d**) Fabrication process of the fiber-shaped OLED and device pictures. (**a**) Reproduced with permission [70]. Copyright 2018, American Chemical Society. (**b**) Reproduced with permission [70]. Copyright 2018, American Chemical Society. (**c**) Reproduced with permission [73]. Copyright 2020, American Chemical Society. (**d**) Reproduced with permission [71]. Copyright 2018, Royal Society of Chemistry.

The most important results about the integration of OLED devices with textiles will be illustrated below, both for single-fiber devices and planar OLEDs (see Table 3 and Table 4). The first OLED on a single fiber was fabricated by O’Connor et al. in 2007 [68]. Specifically, all layers were deposited via thermal evaporation on a polyimide-coated silica fiber, producing devices with an external quantum efficiency (ηEQE) between 0.07% and 0.15%. Despite the low efficiencies and although the device was made on a silica fiber and not on a textile one, the importance of this work lies in the fact that it represents the first successful attempt to deposit an OLED on a fiber substrate. Another OLED fabricated all-around a fiber was developed by Kwon et al. in 2015 [69]. Here, an anode and active layer were deposited through dip coating to coat the fiber concentrically while, for cathode deposition, LiF/Al were thermally evaporated on the emissive layer. With this new configuration, a luminance of 1458.8 cd/m^2^ and a current efficiency (CE) of 3 cd/A were achieved. In 2018, the same group reported another OLED device on a single fiber with an inverted structure [70]. The cathode, the electron injection layer (EIL), and the active material were deposited through dip coating while the hole injection layer (HIL) and the anode were thermally evaporated. A schematization of the fabrication process is shown in Figure 4a. This new configuration exhibited a luminance of 11,780 cd/m^2^ and CE of 11.1 cd/A. Additionally, after encapsulation with a 50-nm-thick Al_2_O_3_ layer, fiber devices showed operating lifetimes of approximately 80 h comparable to the control devices. During the same year, another OLED device on fiber was reported by Ko et al. [71]. The most interesting aspect of this work was the employment of a hollow-core fiber as a substrate. This choice allows for the suppression of the wave-guided light loss at the electrode/substrate interface and avoids the use of additional internal and external light extraction structures (Figure 4d). Thanks to these advantages, the hollow-fiber OLEDs achieved a luminance of 6300 cd/m^2^ and CE of 11 cd/A. Moreover, the insertion of an optically active solution into the empty core of the fiber can be exploited to tune the emission wavelength of the device. 

An important improvement in fiber OLED efficiencies was achieved by Ko et al. in 2020 [72]. In particular, using a hybrid PEDOT:PSS/Ag fiber as a transparent conducting electrode (TCE) embedded in the polymeric substrate, it was possible to fabricate a fiber OLED characterized by a luminance of 4200 cd/m^2^, a CE of 39.6 cd/A, and an EQE of 11.3%, performances comparable to that of the corresponding planar ITO-OLED. In the same year, the fabrication of quantum-dot light-emitting diodes (QLEDs) on a single fiber was reported by Lee et al. [73]. Using CdSe/ZnS and CdS/ZnS core-shell QDs, devices with three different emitting colors (red, green, and blue) were obtained with good performances (340 cd/m^2^ at 13 V—blue QLED, 2044 cd/m^2^ at 10 V—red QLED, 2240 cd/m^2^ at 10 V—green QLED). Furthermore, thanks to the mechanical stability of the fiber, all QLEDs keep 72% lighting at 90° bending, as is visible in Figure 4c. OLED pixels on stripe-shaped PET fibers were also fabricated and were subsequently assembled with conductive fibers to obtain a passive matrix-based textile [74]. The OLED was based on thermal evaporated phosphorescent material as an emitting layer and it showed a maximum current efficiency of about 46 cd/A. Moreover, the woven OLED textile was stable in water and under applied tensile force. The most recent work on fiber OLEDs reports about the deposition of a phosphorescent active layer via dip-coating technique [75]. In particular, three different phosphorescent materials were tested as active layers to prepare phosporescent OLEDs (phOLEDs) with three different emitting colors (red, green, and blue). The resulting devices showed a maximum luminance of 4462, 11,482, and 1199 cd/m^2^ and a CE of 16.3, 60.7, and 16.9 cd/A for red, green, and blue phOLEDs, respectively. Moreover, after encapsulation with a 50-nm-thick Al_2_O_3_ layer, these fiber phOLEDs showed a storage lifetime longer than 4 days and an operating lifetime longer than 10 h.

**Figure 5:**
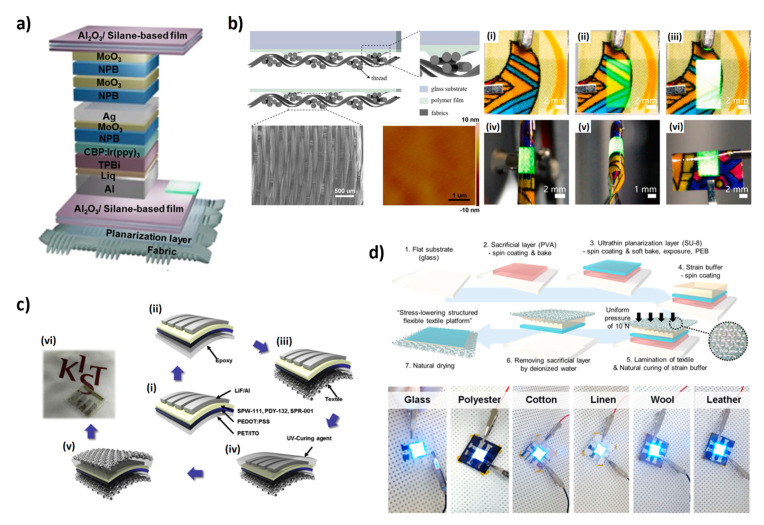
(**a**) Schematic illustration of the fabric-based OLED with planarization and encapsulation multilayer. (**b**) Fabric planarization process by using a polymer film and a glass substrate, SEM picture of the fabric and AFM image of the planarized fabric, photographs of the OLED device on fabric at different driving voltages and under bending conditions. (**c**) Scheme of the fabrication process of a textile/PLED/textile structure. (**d**) Representation of a planarization process based on a replicating method with a sacrificial layer and pictures of OLEDs fabricated on different kinds of planarized textiles. (**a**) Reproduced under the terms of the CC BY 4.0 license [81]. (**b**) Reproduced with permission [83]. Copyright 2020, Elsevier. (**c**) Reproduced with permission [84]. Copyright 2016, Elsevier. (**d**) Reproduced under the terms of the CC BY 4.0 license [82].

As for the fabrication of planar OLEDs on textiles, the first attempt was reported by Janietz et al. in 2013 [76]. Specifically, 1 cm^2^ orange and green OLEDs were integrated into textiles like spacer warp knitting. The green and orange OLEDs showed a brightness of 1900 and 1100 cd/m^2^ and CE of 11.3 and 2.5 cd/A, respectively. Nevertheless, despite the good efficiencies, both OLEDs were not deposited directly on fabric but just integrated after their fabrication. To the best of our knowledge, the first OLED fabricated directly on a fabric substrate was produced by researchers of KAIST in 2013 [77]. An all top-emitting OLED was deposited through thermal evaporation on a plain-woven fabric made of polyester fibers. Before OLED fabrication, the fabric surface was planarized by depositing two polymeric ductile materials, polyurethane (PU) and poly(vinyl alcohol) (PVA), via lamination and spin coating. Thanks to the planarization, a reduction of the roughness from 10 µm to 0.386 µm was reported. OLEDs fabricated on planarized fabric showed a luminance of 7000 cd/m^2^ and CE of around 8 cd/A, comparable with performances of the devices fabricated on glass substrates. In 2014, the same group proposed another OLED device fabricated on textiles with an improved planarization process and a multilayer of 3.5 dyads of aluminum oxide (Al_2_O_3_) and PVA for the encapsulation [78]. They reported a planarization technique employing two layers of PU at low and high viscosity to reduce the large-scale roughness and to ensure a flat surface. The device was obtained via thermal evaporation, and 2 dyads of NPB (N,N′-Di(1-naphthyl)-N,N′-diphenyl-(1,1′-biphenyl)-4,4′-diamine) and WO_3_ were deposited as a capping and protective layer on top of the device. Al_2_O_3_ and PVA were deposited via atomic layer deposition (ALD) and spin coating, respectively. The OLEDs reached a current efficiency of 3 cd/A and luminance of 2000 cd/m^2^ with similar performance after 400 h in ambient air, proving the good performance of the encapsulation layer. The same OLED, with the same encapsulation and planarization layer, was also proposed with a new capping/protective dyad composed by NPB and ZnS [79]. ZnS was selected because can be deposited in situ while developing the OLED, it also acts as a moisture barrier, and, thanks to its refractive index (~2.3), allows for a high light extraction in the final OLEDs. The resulting devices reached a maximum luminance of ~1500 cd/m^2^ and a maximum current efficiency of ~5 cd/A. Moreover, the barrier properties of the encapsulating multilayer (WVTR = 1.8 × 10^−5^ g/m^2^/day at 30 °C and 90% RH) ensured good performances after 3500 h in ambient air. The same technology was also used to develop a bottom emitting OLED by depositing the anode (PEDOT:PSS) and the active layer (Super Yellow) through spin coating, allowing for the device to partially overcome the disadvantages of thermal evaporation, which is expensive and requires a high vacuum [80]. The result is an OLED with a maximum luminous efficiency and maximum power efficiency of 9.72 cd/A and 7.17 lm/W, respectively, which are approximately 20% lower than those found on the glass reference. An OLED based on a phosphorescent emitting material was also fabricated on planarized fabric (Figure 5a) with a luminance of ~93,000 cd/m^2^ and CE of ~49 cd/A [81]. In the study, researchers also reported a new multibarrier encapsulation composed of Al_2_O_3_ and a soft silane-based polymer that provides an excellent optical transmittance (>90% in the visible region) and an outstanding water vapor transmission rate (WVTR) of 10^−6^ g/m^2^/day, nearly identical to that of commercial glass lids. The WVTR of the multilayer barrier remains almost unvaried after 1000 cyclic bends with a radius of 2 cm. Researchers of KAIST also reported a new approach for the fabrication of OLEDs on textiles by using an ultrathin planarization layer and a strain buffer [82]. In particular, they used a new surface-replicating method with a sacrificial layer to obtain a surface with the same flatness of glass. All the phases of the planarization process are shown in Figure 5d. Thanks to this planarization process it is possible to fabricated OLEDs on every kind of textile with performances comparable to that obtained on glass. Additionally, thanks to the Al_2_O_3_/ZnO encapsulation multilayer that showed a WVTR of 7.87 × 10^−6^ g/m^2^/day, the textile OLEDs can retain more than 90% of their luminance after 200 min underwater.

A different approach for fabric planarization was reported by the group of Hong-Bo Sun in 2017 [85]. A commercially available photopolymer was deposited via spin coating on top of a silk substrate, providing a good surface morphology (root mean square (RMS) = 0.682 nm) but also high flexibility and mechanical robustness. The device on top of a planarized silk substrate was deposited via thermal evaporation by using an iridium complex as an emitting material and the OLED showed a maximum luminance and current efficiency of 45,545 cd/m^2^ and 37.7 cd/A, respectively. Additionally, not only were OLED emission results uniform and defectless, but even after 100 bending cycles no deterioration in luminance and efficiency was observed. The same group improved the performance of the planarization layer by using the same photopolymer previously reported but with a new template-stripping deposition process [86]. They fabricated an OLED on the planarized textile, characterized by a luminance of 15,000 cd/m^2^ and CE of around 78 cd/A and outstanding bending stability. The variations of luminance and CE are only 1.7% and 8%, respectively, after 1000 bending cycles at a 1 mm bending radius, which is the best bending stability reported so far. These performances, comparable with those of conventional planar devices, can be ascribed to the flat and smooth planarization layer. They also reported a highly transparent OLED on planarized nylon by using ultrathin metal films as an anode and cathode and by employing a capping layer of NPB [83]. With this new architecture, the device reached a maximum luminance larger than 10,000 cd/m^2^ and a CE of 16.7 cd/A. Unfortunately, due to the small thickness of the metal electrodes (7 nm of Au anode and 9 nm of Ag cathode), the OLED has poor bending stability and degrades quickly after few bending cycles.

An innovative and environmentally friendly textile substrate for OLED integration was developed by Park et al., and it was based on a keratin/PVA nanofiber mixture [84]. The fabric exhibited high optical transparency (transmittance of ~85% at λ = 550 nm) and surface roughness with an RMS of 142.7 nm and an arithmetical mean height (Ra) of 104.2 nm. OLEDs fabricated on PET were sandwiched between two sheets of biocompatible fabric (Figure 5c) and in these conditions the device exhibited a luminance of 2781 cd/m^2^, 2430 cd/m^2^, and 6305 cd/m^2^ and maximum current efficiency of 0.29 cd/A, 0.10 cd/A, and 0.38 cd/A for white, red, and yellow emissions, respectively. Although the device was not directly fabricated on top of the textile substrate, this result is interesting for the future development of eco-friendly textile electronic devices.

A well-performing encapsulation process for textile-based organic device fabrication was recently proposed by Jeong et al. [87]. The encapsulation consists of a nano-stratified barrier with an SiO_2_ polymer composite, and it was obtained by using ALD and spin-coating techniques. A polymeric solar cell and an OLED were fabricated on the barrier-coated textile, and they preserved almost the same initial performances after several washing tests. 

Not only simple OLED devices but also active-matrix organic light-emitting diodes (AMOLEDs) can be integrated with textiles and such technology is particularly promising for display applications. Kim and Song proposed an AMOLED on textile device in 2016 [88]. Both organic thin film transistors (OTFTs) and OLEDs were fabricated on PET fabric previously planarized by using polyurethane (PU) and photo-acrylic (PA) films. All OTFT and OLED layers were evaporated and patterned by a lift-off process, except for the OTFTs’ active material (specifically, TIPS-pentacene), which was inkjet-printed. As result, the OTFT mobility was 0.34 cm^2^/V and the OLED luminance was 64,459 cd/m^2^ at 12 V. Kim and Song recently reported another AMOLED device with improved performance [89]. A high mobility of 0.98 cm^2^/V was achieved by using carbon nanotube/Au electrodes and the photo-acryl as a dielectric. Moreover, a protective layer composed of PVA and PA was used to improve the stability of devices. Indeed, after 20 days in air, the luminance of the encapsulated device decreased to 64%, while that of the bare device decreased to 54%.

In conclusion, OLEDs are well-performing light-emitting devices for textile integration. Nonetheless, some practical aspects need to be fully addressed for commercial application. Indeed, the performance and manufacturing costs of the encapsulation barrier layer represent, at the moment, the main challenge.

### 2.4. LEC Devices

Light electrochemical cells (LECs) are an evolution of OLED devices; in the mid-1990s *Pei* and co-workers discovered such new technologies by modifying the emissive layer of a polymeric OLED by adding mobile ions and a solid electrolyte and they explained the charge injection from the electrodes as a redox electrochemical process [91]. In addition to conjugate polymers, this kind of phenomenon was observed also for ionic transition metal complexes, so we can distinguish between pLECs (polymeric LEC) and iTMC-LECs (ionic transition metal complexes LECs) [92]. When an external bias is applied, the electrical field induces a redistribution of the ions; in particular anions and cations migrate towards the corresponding electrodes with the formation of an electric double layer (EDL) at the interface between the electrode and the active layer. In particular, when the applied voltage is low, the carrier injection from the electrodes is limited, the ions drift is predominant and induces the formation of the EDL, while the central part of the active layer remains field-free; in these conditions, the device shows low luminance values. When the bias increases, more electrical charges are injected into the organic layer, inducing redox processes. The new charge species are stabilized by the ions located at the organic layer/electrode interface with the formation of doped zones; the result is analogous to a pin OLED structure (Figure 1d) [92]. The doping can improve the charge injection from the electrodes because it induces a band bending of the HOMO (Highest Occupied Molecular Orbital) and LUMO (Lowest Unoccupied Molecular Orbital) levels towards the Fermi level. Considering the improved charge injection, every kind of electrode can be used in LEC structure without concerns related to the work function level. The architecture of a LEC device is very simple and includes an active layer (composed of electroluminescent material, ions, and a solid electrolyte) sandwiched between two electrodes, of which at least one is transparent (Figure 1d). The active layer can be easily deposited by solution processes and higher thicknesses can be used with an improvement in the mechanical stability and less concerns related to short circuits; high stable and non-reactive electrodes can be used without degradation problems. On the other hand, if the in-situ doping process is not properly controlled, the doped regions may continue to grow towards each other until they meet at the center of the device. This effect causes a quenching process and a drastic reduction of luminance. The migration of the doped zones is strongly related to the electronic and ionic mobility of the active layer, the applied bias, and the thickness of the active layer. All of these factors should be evaluated to optimize device performances. The ionic mobility also influences the switch on voltage and time. The latter can assume values between few milliseconds to several hours and for such reason, LEC technology is not suitable for display applications.

**Figure 6:**
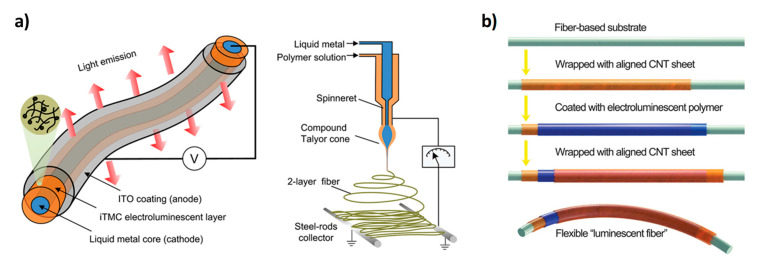
(**a**) Architecture of an electroluminescent fiber based on an ionic transition-metal complex (iTMCs) and scheme of the co-electrospinning setup. (**b**) Scheme of the fabrication process of a fiber-based-LEC. (**a**) Reproduced with permission [93]. Copyright 2012, American Chemical Society. (**b**) Reproduced with permission [95]. Copyright 2015, Royal Society of Chemistry.

Some attempts to integrate this technology with fabrics have been carried out in recent years, although the results are still limited and not comparable with those achieved by the other technologies already discussed. In 2012, a single electroluminescent fiber based on an iTMC was developed by using a co-electrospinning technique as reported in Figure 6a [93]. The device architecture is also illustrated in Figure 6a and consists of a Galinstan liquid metal core, an iTMC based electroluminescent layer, and an ITO coating. The fiber-based light-emitting device showed a turn-on voltage of 4.2 V, uniform light emission, and has great potential for textile integration thanks to its flexibility, conformability, and lightweight. A different approach for the fabrication of LEC devices on single fiber was developed by the group of H. Peng [94,95]. Starting from a metal wire-based cathode, ZnO and a polymeric electroluminescent layer were deposited by dip coating and finally, a carbon nanotube sheet wrapped around the coated wire was used as a transparent anode [94]. The CNT sheet was obtained by a dry-drawing process starting from a CNT array synthesized by chemical vapor deposition; it shows high transmittance and electrical conductivity around 102–103 S/cm which remain almost unvaried after 1000 bending cycles. The device reached a luminance value of about 600 cd/m^2^ and an efficiency of 0.83 cd/A. The fiber-based LEC device was also fabricated by using the CNT sheet both as cathode and anode layer as you can see in Figure 6b; in this case, smaller luminance and efficiency values (505 cd/m^2^ and 0.51 cd/A) were reported compared to the previous device with the CNT sheet only as a cathode [95]. A LEC device was also developed as a planar device on textile by using a spray-coating deposition [96]. The fabric was composed of conductive and polymeric fibers embedded in a polyurethane matrix and the thickness of the PU matrix was designed to have conductive bumps on the surfaces as electrical contact of the light-emitting device. On top of the textile, PEDOT:PSS, active layer, and aluminum were deposited to fabricate the device that showed high luminance (>4000 cd/m^2^) and good efficiency (3.4 cd/A). Spray coating was also employed to obtain all solution-based LECs (bottom electrode—active layer—top electrode) on standard polyester cotton textile previously planarized by using a UV curable PU resin [97]. The obtained performances are poor, but this result is really interesting for the development of all solution-based devices toward a low-cost fabrication process.

As previously mentioned, the possibility to use high stable electrodes without charge injection problems is a great advantage for this kind of light-emitting architecture, compared to OLED devices. In particular, silver electrodes can be easily obtained by solution processes by using silver nanoparticles or nanowires with the possibility to fabricate all solution-based low-cost light-emitting devices on textile and other kinds of innovative substrates [98,99,100]. On the other hand, further efforts are needed to optimize the electro-optical characteristics of LEC devices.

### 2.5. POFs

Since the 1960s, optical fibers have been known for light transmission and communications [101]. Optical fibers are very interesting for wearable applications thanks to their similarity with traditional textile fibers, and for such reason they can be processed for fabric weaving as standard textile yarns [102]. In particular, polymeric optical fibers (POFs) are very promising for textile integration thanks to their flexibility, light weight and low cost, although they do not reach the performance levels of traditional glass fibers in terms of propagation losses and data transmission capacity [102]. A typical optical fiber is composed of a central region, the core, and by an external region, the cladding; the refractive index of the core is higher than that of the cladding. The POF is fabricated by two principal techniques: drawing form preforms and extrusion [103], and typically employed polymers for their fabrication are PMMA, polystyrene, and polycarbonates for the core, while the cladding is usually composed of fluorinated polymers [104]. A POF is a cylindrical dielectric waveguide usually employed to transport light between the two ends of the fiber by exploiting the total internal reflection phenomenon (distal-end-emitting optical fibers). Side emission can occur in specific conditions and such technology can have great opportunities for large-area lighting. Two main approaches are usually employed to obtain side emission: microperforations of the fiber or macrobending, as shown in Figure 1e [102]. Indeed, the microperforation and the damaging of the cladding induce light scattering and leakage through the cracks. Microperforation can be obtained by using different methods: notching [105,106], abrasion [105,106], laser micromachining [107,108], surface chemical etching [106,109], etc. In 2003, Harlin et al. obtained light-emitting textiles with a conventional weaving machine by using polycarbonate fibers, and different mechanical methods were tested to induce microperforations [105]. Konkar et al. compared a mechanical and a chemical treatment to obtain side-emitting POFs, and it was demonstrated that the chemical etching ensures better control [109]. In general, mechanical methods are considered more aggressive since they can cause damage to the fibers with a weakening of the core structures. To improve the robustness after mechanical treatments, polymeric overcoats can be deposited [106]. Anyway, mechanical treatments cannot be precisely controlled and they are not reproducible, as a result, the light distribution is not uniform and different from sample to sample. The laser treatment is more precise than mechanical and chemical treatments and it can also be employed to obtain specific patterns, as reported by Shen et al. [107]. Side emission from optical fibers can also be obtained via macrobending in a way to have a propagation angle higher than the critical angle and light emission out of the fiber. Different from the microperforation approach, no modification is necessary to the structure of the optical fibers, and it is sufficient to design specific embroidery or weaving architectures during the textile manufacturing to obtain side emission. In particular, the absence of post-treatment of the fiber can be considered an important aspect for the reduction of the cost. On the other hand, the intensity of the light emission in the macro-bending approach is very sensitive to the bent radius and it is necessary to ensure that a constant bending radius is maintained in the whole textile also when it is worn. For this purpose, Wang et al. reported a theoretical equation to quantify the correlation between the light emission efficiency and the bending radius of POFs [110]. Although it is essential to control the bending radius of the fiber to have a good lateral emission, it is important to avoid abrupt folding or bending of the fiber that can cause breakage and affect the light emission uniformity. To avoid the risk of breakage, weaving is usually preferred compared to knitting. Therefore, with the appropriate precautions, side-emitting POFs can be processed to form any type of fabric in combination with traditional yarns, and the result is a textile with good tactility and comfort. To obtain light-emitting textiles from lateral-emitting POFs, they are connected to external light sources together with a power source and a motherboard. LEDs are usually employed as lightweight light sources that can be connected at each end of the fiber to obtain an intense and uniform light emission. Indeed, if the LED is connected only at one end of the fiber, the light emission decreases progressively along the fiber. The motherboard can also be designed to incorporate sensors and programs to obtain different illumination and interactive effects. All these external elements are small and lightweight, and they do not affect the portability of the garments. Differently from light-emitting textiles obtained using LEDs, the light emission from POFs is not point-like and so it is possible to have a large area of emission. On the other hand, the emission area is not uniform as it is for planar light-emitting devices fabricated on textiles. Anyway, side-emitting POFs represent a robust, stable, and low-cost technology for light-emitting textiles that is already on the market.

## 3. Health and Environmental Concerns

The integration of electroluminescent devices with textiles raises some issues related to health and environmental concerns that should be considered for correct and safe use. 

The ACEL devices are among the safest electroluminescent devices in terms of their chemical composition. Biocompatible polymers such as polyvinylpyrrolidone (PVP) and PDMS are largely employed as dielectrics in ACEL devices. As for phosphors, they are composed of a host material and a dopant. Typically, hosts are oxides, nitrides, sulfides, or selenides of zinc, cadmium, manganese, aluminum etc. Although some of these compounds can be toxic, copper-doped ZnS is the most used one, and is currently used in glow-in-the-dark toys.

Regarding LEDs, they can contain arsenic, gallium, indium, antimony etc. which can cause human and ecological toxicity effects [111]. Researchers from the University of California tested the toxicity of different kinds of LEDs by using a standardized leachability test and they observed that all the above-mentioned elements are below the threshold limit, according to California State regulations, while copper, lead, and nickel are above legal limits [112]. Such metals are usually employed in ancillary technologies such as wires, solders, glues etc., and they represent the main contribution to the total hazard potential. Moreover, such ancillary technologies are employed in all the device architectures mentioned in this review and this aspect should be considered in terms of potential toxicity.

As for OLED devices, a recent study compared the environmental impact of OLED technology with specific concern given to displays [113]. Of course, such a kind of application is quite different form the e-textile one, but it provides some indications. In particular, it was reported that silver, chromium, beryllium, and copper exceed legal limits in a leachbility test. Unfortunately, most of organic materials used in OLED and other organic-based light-emitting technologies (e.g., LECs) have not yet been tested and further studies are necessary to assess their environmental and health impacts.

No reports are available about the effect of such devices to human health. Anyway, all of them need an appropriate packaging and encapsulation to protect them from environmental agents and mechanical stress so there is no direct contact between the device and the wearer. Any effects related to the diffusion of potentially dangerous materials toward the skin could be then eliminated and/or minimized.

In light of the considerations reported, all the electroluminescent devices must be disposed in an appropriate way at the end of their life as they are potentially harmful to the environment.

## 4. Applications

**Figure 7:**
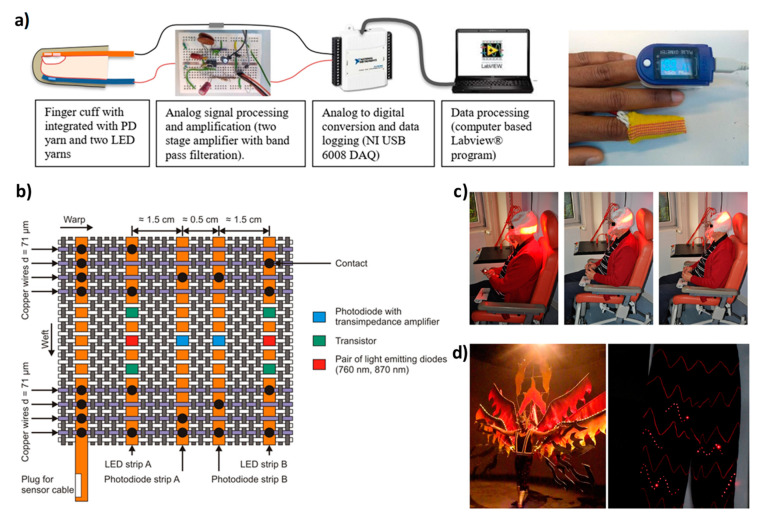
(**a**) Scheme of the PD/LED yarn system for heart rate detection and picture of the comparison between the e-yarn system and a commercial one. (**b**) Schematic representation of a textile-based sensor with two photodiode strips, two LED strips, and a bus bar strip, for near-infrared spectroscopy. (**c**) Picture of a photodynamic therapy session by using a POF-based light-emitting textile at 635 nm. (**d**) Carnival costume integrated with LEDs. (**a**) Reproduced under the terms of the CC BY 4.0 license [121]. (**b**) Reproduced with permission [120]. Copyright 2013, The Optical Society. (**c**) Reproduced under the terms of the CC BY 4.0 license [137]. (**d**) Reproduced under the terms of the CC BY 4.0 license [116].

The integration of light-emitting devices with fabrics is interesting for numerous applications in different areas. Light-emitting clothes can be used for the fashion industry and artistic exhibition but also for visual communications such as promotional events and visibility improvement in low-light conditions. Light emission gives a new look to our clothes and at the same time can be exploited to communicate with viewers. Both academia and industry are working in this application field. Shenzhen Fashion Luminous Technology Co. Ltd. is a Chinese company that was established in 2013 and it is specialized in the manufacturing of light-emitting optical-fiber-based fabrics and clothing. DreamLux is an Italian company that developed a light-emitting textile (LumiGram^®^) based on side-emitting optical fibers and it provides ready-for-use “Luminous Panels” composed of a piece of fabric (optical and traditional fibers), LEDs, and a power supply. Another example of light-emitting clothes was reported by Philips in 2006; Lumalive is a technology based on flexible LED arrays located beneath the outer fabric. This lighting system does not compromise the softness of the cloths and it can be easily removed when you want to wash the garments. The American startup Lumenus developed smart clothes and other accessories for runners and cyclists based on LEDs. A similar product was created by researchers at the Taiwan Textile Research Institute in collaboration with PEGA D&E and it consists of a cycling jacket integrated with LED yarns (LaightFairy). CuteCircuit is another company that has created many illuminating costumes based on LEDs. Recently, Tan explored the challenges related to the designing of POF-based textiles for the creation of dynamic fashions [114]. In the same field, researchers of electronic engineering and fashion design departments at Kookmin University published a study where they reported some technical and design guidelines for LED integration into textiles and they also developed some prototypes [115]. Hardy et al. demonstrated the possibility of integrating, via embroidering technique, a non-stretchable LED yarn with stretch fabric for a carnival costume (Figure 7d) [116]. A digital watch display [117] and a matrix display [118] on fabric were also fabricated by exploiting the ACEL architecture and screen-printing deposition technique.

Sensing is another possible application area for light-emitting textiles as both input and output elements. Textile is the closest layer to our body, and for such reason it is the ideal substrate for sensor integration for physiological monitoring; at the same time, clothes are ubiquitous in everyday life and they can be used as supports for portable environmental sensors useful to preserve our health and our quality of life. Cherenack et al. reported in 2010 on a combination of temperature and humidity sensors with LEDs as visual indicators on textile [51]. A prototype of smart clothing designed for construction worker safety was demonstrated in 2015. It consists of a temperature sensor with a light emission (LED) and an acoustic alerting system when abnormal temperatures are recorded [119]. In 2017, Liu et al. reported a textile pressure sensor for monitoring physiological signals and human motion with a real-time visual response provided by an LED [2]. LEDs have also been integrated with photodiodes for arterial oxygen saturation (Figure 7b) [120] and heart rate [121] monitoring (Figure 7a). POFs can also be used as sensors by exploiting two different approaches: wavelength modulation or intensity modulation. Bragg-grating-based fibers are usually employed for wavelength modulation. When an incident broad band light enters the gratings, a specific wavelength is reflected that is equal to 2nΔ, where n is the refractive index and Δ is the grating pitch [102]. As a consequence of external signals such as temperature and strains, both the n and Δ change and so the reflected wavelength and its variation can be exploited in sensing applications [122,123,124]. Regarding the intensity modulation, in this case the light intensity can be lost by defects and geometrical perturbation of the fiber axis and it can be related to external signals that can be monitored [124]. POF-based sensors have been largely employed for monitoring of breathing [125,126,127,128], plantar pressure and temperature monitoring [129], and monitoring of oxygen saturation of hemoglobin [130]; more details can be found in different reviews [102,104,131].

Finally, light-emitting textiles can be used for light therapy. Indeed, light has been largely applied in clinical practice for disease treatments, pain relief, tissue repair, and so on [132,133]. Textiles can improve user acceptance thanks to their conformability and the possibility to fabricate portable light-emitting fabrics. Furthermore, since the textile is very close to the skin, this reduces the amount of light that is lost and makes the treatment more effective. In 2013, a textile based on side-emitting POFs obtained via weaving was tested during in vitro experiments that demonstrated a significant increase in collagen production in human fibroblasts irradiated by the fabric [134]. Koncar worked on the development of textile light diffusers by exploiting the macro-bending of POFs with homogenous light emission and flexibility, obtaining suitable performances for photodynamic therapy [135,136], and such technology has been recently tested both during in vitro and in vivo experiments [137]. LEDs have also been integrated with textiles for light therapy; researchers from Ghent University demonstrated an LED-based textile that delivers breathability, excellent mechanical compliance, and good irradiance without excessive thermal heating [138]. All the applications illustrated here can greatly improve the quality of our lives and so all the research results will be essential for future development and improvement.

## 5. Conclusions

Light emission is a very interesting functionality that can be integrated with textiles for applications in different fields: fashion and artistic performances, visual communications, sensing, and light therapy. Different technologies can be useful for this purpose, and they have been reported in this review. Light-emitting devices can be directly fabricated on fibers and planar textiles in the form of ACELs, OLEDs, and LECs. 

Although electroluminescent fibers could in principle be employed directly by using weaving, knitting, and embroidering techniques, the fabrication on a single fiber requires the preparation of numerous devices and the design of a specific production line. Moreover, it is fundamental that the fibers are able to withstand the mechanical stress required by fabric processing. On the other hand, the manufacturing of planar electroluminescent textiles is preferable from a practical and economical point of view. Indeed, large-area planar devices can be developed directly on textiles in a single-step process, such as for devices on top of standard flexible substrates (e.g., PET). In this case, a further planarization step could be necessary to reduce surface roughness and to ensure proper operation conditions.

Of course, each device typology has positive aspects and disadvantages. ACEL devices can be easily deposited via solution-based processes by using commercially available inks. Different highly scalable deposition techniques have been developed with good results, such as screen printing, slot-die coating, dispensing printing, etc. Unfortunately, such devices show a maximum luminance of few hundreds cd/m^2^ and a very high driving voltage (higher than 100 V) is usually employed; in particular, this last aspect can be considered hazardous for wearable applications. 

Textile-based OLEDs show very high luminance and current efficiency and they are fabricated by using both thermal evaporation and solution-based deposition processes. However, thermal evaporation is the most used deposition technique, although it is expensive and not scalable. For such reasons, all solution-based OLED devices are a very hot topic in this field. Planarization and encapsulation represent two critical issues that must be addressed for the future development of this technology. Different kinds of planarization processes have been developed based on various polymeric materials. Encapsulation is necessary to avoid the reaction between the device and external agents (e.g., water and oxygen) that cause degradation. Typical flexible encapsulation consists of a multilayer of inorganic and organic materials deposited via atomic layer deposition (ALD) and solution processes, respectively. WVTR values close to that of glass lids (10^−6^ g/m^2^/day) have been achieved on textiles with good stability results. 

LECs have a device architecture similar to that of OLEDs but with two important differences: the device thickness is higher than that of OLED and so there are less concerns about short circuits; the work function of electrodes is not important for the charge injection and so high stable electrodes can be used so the entire device is also more stable. Despite these advantages over OLED devices, LECs do not show the excellent electro-optical performances reported for OLEDs, and only few examples of textile-based LECs are reported in the literature.

Inorganic LEDs are another option for the development of light-emitting textiles, but in contrast to OLEDs they are rigid and point-like devices and for such reason they are not the ideal candidates. At the same time, LEDs show very high performance and stability and this technology is commercially available. LEDs have been integrated with textiles by simply attaching them on textile-based electrical circuits or by embedding the device into the fiber, improving the mechanical robustness of the e-textile.

All the mentioned technologies consist of light-emitting devices integrated directly with textiles; however, it is possible to convey the light inside the fabric using polymeric optical fibers. Side emission can be obtained by using two different approaches, microperforation and macrobending. These fibers, coupled with LEDs, can be integrated with traditional fibers to obtain light-emitting textiles. LEDs and POFs are the most mature technologies among those mentioned in this review and they are already used in real applications. In any case, research is very active in this field and there will be important progress in the near future.

## Figures and Tables

**Table 1:** Deposition techniques, structure, and performances of ACEL devices on a single fiber.

Year	Deposition Techniques	Device Structure	Performances	Ref.
2012	Automatic dispensing and curing process	Silver-coated yarn/DuPont dielectric paste/DuPont phosphor ink/silver-coated yarn	1.3 cd/m^2^ at 370 V and 2 kHz	[21]
2017	Slot-die coating	Silver-coated yarn/DuPont dielectric paste/DuPont phosphor ink/silver-coated yarn	50 cd/m^2^	[22]
2017	Slot-die coating	Silver-coated yarn/DuPont dielectric paste/DuPont phosphor ink/DuPont conductive paste	n.a.	[23]
2017	Dip coating	PET fiber/AgNWs/silicone/phosphor/AgNWs/silicone	202 cd/m^2^ at 195 V and 2 kHz	[25]
2018	Slot-die coating	Silver-coated yarn/DuPont dielectric paste/DuPont phosphor ink/DuPont conductive paste	50 cd/m^2^ at 100 V and 20 kHz	[24]
2018	Dip coating	PDMS fiber/AgNWs/phosphor:PDMS/AgNWs	100 cd/m^2^ at 400 V and 1 kHz	[27]
2018	3D printing and automatic wrapping	Elastic polymer fiber/Aligned CNT sheet/Silicone elastomer/Light-emitting layer/Aligned CNT sheet	15 cd/m^2^ at 6.4 V/μm and 1.5 kHz	[28]
2018	Extrusion	Two inner hydrogel electrodes + ZnS:silicone elastomer	250 cd/m^2^ at 8 V/μm and 1.5 Hz	[29]
2018	Spin coating	Graphene/ZnS:Cu/BaTiO3/Ag	n.a.	[30]
2019	Dip coating	PVA fiber/AgNWs/ZnS:Cu+PVP/AgNWs	100 cd/m^2^ at 300 V and 0.4 kHz	[26]

**Table 2:** Deposition techniques, structure, and performances of ACEL devices on fabric.

Year	Deposition Techniques	Device Structure	Performances	Ref.
2011	Inkjet printing	PEDOT:PSS/Phosphor:epoxy resin/aluminium	44 cd/m^2^ at 400 V and 0.4 kHz	[34]
2012	Inkjet printing + nozzle extrusion	CNT and/or PEDOT:PSS/dielectric layer/phosphor layer/aluminium	70 cd/m^2^ at 200 V and 3.5 kHz	[35]
2014	Screen printing	Silver/dielectric layer/luminophore/CNT-GNP electrode	n.a.	[36]
2016	Dispenser printing	FabInks bottom electrode/FabInks dielectric/FabInks phoshor/ FabInks transparent tonductor	300 cd/m^2^ at 370 V and 1 kHz	[33]
2017	Chemical deposition + casting	Polypirrole/ ZnS:Silicone elastomer/hydrogel film	350 cd/m^2^ at 5 V/μm and 2 kHz	[38]
2018	Solution-based metallization + spin coating	Gold-coated textile/BaTiO3+PDMS/ZnS:Cu+PDMS/PEDOT:PSS	n.a.	[39]
2019	Embedding of fibers into phosphor:PDMS composite	Ag-coated fibers/ZnS phosphor+PDMS	35 cd/m^2^ at 1.8 V/μm and 2 kHz	[31]
2019	Screen printing	Graphene-based electrode/BaTiO3/ZnS:Cu/BaTiO3/CNT or ATO transparent electrode	300 cd/m^2^ at 160 V and 2 kHz	[37]
2020	Solution-based metallization + spin coating + lamination	Gold-coated textile/ZnS:Cu+Ecoflex/gold-coated textile	n.a.	[32]

**Table 3:** Structure and performance of OLEDs on fiber.

Year	Deposition Techniques	Device Structure	Performances	Ref.
2007	Thermal evaporation	Al/Ni/CuPc/NPD/Alq_3_/LiF/Al	η_EQE_ 0.07 ÷ 0.15% at 0 ÷ 10 V	[68]
2015	Dip coating + thermal evaporation	PEDOT:PSS/Super Yellow/LiF/Al	1458.8 cd/m^2^at 10 V 3 cd/A at 6 V	[69]
2018	Dip coating + thermal evaporation	PEDOT:PSS/ZnO NPs/PEI/Super Yellow/MoO_3_/Al	11,780 cd/m^2^ at 10 V 11.1 cd/A at 5 V	[70]
2018	Thermal evaporation	ITO/2-TNATA/NPB/Alq_3_/LiF/Al	6300 cd/m^2^at 13 V 11 cd/A at 12 V	[71]
2020	Spin coating + thermal evaporation	Hybrid fiber TCEs/PEDOT:PSS/PVK:TPD:PBD:Ir(mppy)_3_/TPBi/LiF/Al	4200 cd/m^2^ at 12 V, 39.6 cd/A and 11.3% of EQE at 7 V	[72]
2020	Dip coating + thermal evaporation	PEDOT:PSS PH1000/PEDOT:PSS AI4083/TFB/QDs/AlZnO/Al	340 cd/m^2^ at 13 V for CdS/ZnS (blue QLED) 2044 cd/m^2^ (red QLED) and 2240 cd/m^2^ (green QLED) at 10 V for CdSe/ZnS	[73]
2020	Thermal evaporation	ITO/HAT-CN/TAPC/TCTA:Ir(ppy)_2_acac/B3PYMPM/Liq/Al	2900 cd/m2 at 5 V, 46 cd/A at 2.4 V	[74]
2021	Dip coating + thermal evaporation	PEDOT:PSS/ZnO NPs/PEI/PVK:26DCzppy:Ir(ppy)3 (30:30:1 weight ratio)/TCTA/MoO3/Al—green	11,482 cd/m^2^ at 6.5 V 60.7 cd/A at 4.5 V for the green OLED	[75]
		PEDOT:PSS/ZnO NPs/PEI/PVK:TPBi:Hex-Ir(phq)2acac (25:25:1 weight ratio)/TCTA/MoO3/Al—red	4462 cd/m^2^ at 7 V 16.3 cd/A at 4.5 V for the red OLED	
		PEDOT:PSS/ZnO NPs/PEI/PVK:26DCzppy:Ir(Fppy)3 (30:30:1 weight ratio)/TCTA/MoO3/Al—blue	1199 cd/m^2^at 6 V 16.9 cd/A at 4 V for the blue OLED	

**Table 4:** Structure and performance of OLEDs on textiles.

Year	Deposition Techniques	Device Structure	Performances	Ref.
2013	Thermal evaporation	Ag/WO_3_/NPB/Alq_3_/Liq/Al/Ag/NPB	7000 cd/m^2^ and 8 cd/A at 6 V	[77]
2014	Thermal evaporation	Al/Liq/Alq_3_/NPB/WO_3_/Ag	2000 cd/m^2^at 7.5 V 3 cd/A at 6.5 V	[78]
2015	Spin coating + thermal evaporation	PEDOT:PSS/Super Yellow(PDY-132)/LiF/Al	5000 cd/m^2^ at 6 V. 9.72 cd/A at 5.5 V7.17 lm/W at 4 V.	[80]
2015	thermal evaporation	Ag/HAT-CN/NPB/TAPC/CBP: Ir(ppy)_3_/TPBi/LiF/Al	64,459 cd/m^2^ at 12 V	[88]
2016	Spin coating + thermal evaporation	ITO/PEDOT:PSS/emission polymer(SPW-111, PDY-132, and SPR-001)/LiF/Al	2781cd/m^2^ at 13 V 0.29 cd/A at 13 V for the white OLED; 2430cd/m^2^ at 10 V 0.10 cd/A at 10 V for the red OLED; 6305 cd/m^2^at 11 V 0.38 cd/A at 11 V for the yellow OLED	[84]
2016	Thermal evaporation	Al/Liq/Alq_3_/NPB/WO_3_/Ag	1500 cd/m^2^ and 5 cd/A at 8.5 V	[79]
2017	Thermal evaporation	Ag/MoO_3_/NPB/mCP: Ir(ppy)3(6% wt)/TPBi/Ca/Ag	45,545 cd/m^2^ at 10.5 V 37.7 cd/A at 7.5 V	[85]
2017	Thermal evaporation	Al/Liq/TPBi/CBP: Ir(ppy)_3_(8% wt)/NPB/MoO_3_/Ag	93,000 cd/m^2^at 14 V 49 cd/A at 12 V	[81]
2018	Spin coating + thermal evaporation	Ag/PEDOT:PSS/Super Yellow/Ca/Ag	n.a.	[90]
2019	Thermal evaporation	Al/Liq/Bebq_2_:Ir(piq)_3_/NPB/MoO_3_/Ag	1660 cd/m^2^ and 19 cd/Aat 8.71 mA/cm^2^	[87]
2019	Thermal evaporation	Ag/HAT-CN/NPB/TAPC/CBP: Ir(ppy)_3_/TPBi/LiF/Al/Ag	23,673 cd/m^2^at 7 V (bare OLED)16,636 cd/m^2^ at 7 V (encapsulated OLED)	[89]
2020	Thermal evaporation	Ag/MoO_3_/NPB/CBP: Ir(bt)_2_(acac)/TPBi/Ca/Ag	15,000 cd/m^2^at 8 V 78 cd/A at 6 V.	[86]
2020	Thermal evaporation	Au/MoO_3_/NPB/mCP: Ir(ppy)_3_(6% wt)/TPBi/Ca/Ag	17,900 cd/m^2^ at 10 V 12.4 cd/A at 4 V (anode side); 15,300 cd/m^2^ at 10 12.8 cd/A at 4V (cathode side); total current efficiency of the transparent OLED 25.2 cd/A at 4 V	[83]
2020	Thermal evaporation + spin coating	NPB/Ag/MoO_3_/NPB/Alq_3_/Liq/Al ZnS/Ag/ZnO/PEI/ Super Yellow(PDY-132)/MoO_3_/Ag/NPB	About 7000 cd/m^2^ at 5.5 V (OLED); 10,000 cd/m^2^ at 5.5 V (PLED)	[82]

## Data Availability

Not applicable.
